# Role of the Renin Angiotensin Aldosterone System in the Pathogenesis of Sepsis-Induced Acute Kidney Injury: A Systematic Review

**DOI:** 10.3390/jcm12144566

**Published:** 2023-07-08

**Authors:** Sedra Tibi, Garbel Zeynalvand, Hina Mohsin

**Affiliations:** School of Medicine, California University of Science and Medicine, Colton, CA 92324, USA; tibis@cusm.org (S.T.); zeynalvandg@cusm.org (G.Z.)

**Keywords:** sepsis, acute kidney injury, inflammation, oxidative stress, endothelial damage, microthrombi, RAAS, ACEi, ARB

## Abstract

Background: Sepsis is a life-threatening condition responsible for up to 20% of all global deaths. Kidneys are among the most common organs implicated, yet the pathogenesis of sepsis-induced acute kidney injury (S-AKI) is not completely understood, resulting in the treatment being nonspecific and responsive. In situations of stress, the renin angiotensin aldosterone system (RAAS) may play a role. This systematic review focuses on analyzing the impact of the RAAS on the development of S-AKI and discussing the use of RAAS antagonists as an emerging therapeutic option to minimize complications of sepsis. Methods: Studies were identified using electronic databases (Medline via PubMed, Google Scholar) published within the past decade, comprised from 2014 to 2023. The search strategy was conducted using the following keywords: sepsis, S-AKI, RAAS, Angiotensin II, and RAAS inhibitors. Studies on human and animal subjects were included if relevant to the keywords. Results: Our search identified 22 eligible references pertaining to the inclusion criteria. Treatment of sepsis with RAAS inhibitor medications is observed to decrease rates of S-AKI, reduce the severity of S-AKI, and offer an improved prognosis for septic patients. Conclusion: The use of RAAS antagonists as a treatment after the onset of sepsis has promising findings, with evidence of decreased renal tissue damage and rates of S-AKI and improved survival outcomes. Registration: INPLASY202360098.

## 1. Introduction

Sepsis is a major issue that has affected the history of healthcare and, to this day, remains a major cause of morbidity and mortality in the modern world. This life-threatening condition is characterized by a systemic inflammatory response, understood as a dysregulated immunological balance of anti- and pro-inflammatory factors. Major concerns about sepsis arise from system-wide organ damage and possible progression to septic shock, a state of profound circulatory and metabolic failure with an increased risk of mortality. Most hospitals use the systemic inflammatory response syndrome (SIRS) criteria to identify sepsis, which includes two or more of the following: temperature >38 °C or <36 °C, heart rate above 90/min, a respiratory rate greater than 20/min, PaCO2 less than 32 mm Hg, and/or elevated or severely decreased white blood cell count or >10% immature bands. Since these signs may be present in unaffected individuals or lack symptoms in affected cases, the Sepsis-3 Conference in 2016 introduced the quick SOFA (qSOFA) diagnostic. This definition includes two or more of the following: altered mentation, systolic blood pressure of 100 mmHg or less, and/or respiratory rate of 22/min or greater [1]. The progression of sepsis to septic shock occurs when vasoactive therapy is required to maintain a mean arterial pressure ≥65 mmHg and a persistent lactate level of ≥2 mmol/L despite fluid resuscitation [1]. Essentially, these criteria encompass the major concern of sepsis: profound hypotension that puts these patients at risk for organ hypoperfusion and injury.

The overstimulation of a host’s immune system in response to infectious microorganisms such as bacteria, viruses, or fungi is the most attributable cause of sepsis, with diarrheal diseases and upper respiratory infections being the top two causes of sepsis in 2017 [2]. Although the recognition of pathogens is critical for infection control, this inflammatory activation response is responsible for the pathological mechanisms observed in sepsis. Pathogen recognition receptors (PRRs) recognize common metabolic products of microorganisms referred to as pathogen-associated molecular patterns (PAMPs), which include bacterial lipopolysaccharides, lipoproteins, flagellin, and viral genetic material. When activated by PAMPs, PRRs initiate a cascade for an appropriate immune response, including the release of pro-inflammatory cytokines such as interleukin (IL)-1, IL-6, and tumor necrosis factor (TNF)-α [3]. These inflammatory mediators exert effects mainly on endothelial cells to activate vasodilation, coagulation, and leakage to extravasate neutrophils and other inflammatory factors. When unregulated by natural anti-inflammatory and anticoagulant factors, excessive endothelial changes lead to hypotension, a hypercoagulable state, and other characteristics seen in sepsis. While sepsis is likely the result of an infectious agent, not all infections lead to sepsis. Among those with suspected infection, risk factors associated with an increased likelihood of progression to sepsis include older age, abnormal vital signs such as elevated heart rate and respiratory rate, and underlying comorbidities including diabetes mellitus, heart disease, and chronic kidney disease (CKD) [4].

The global number of incident cases of sepsis between 1990 and 2017 indicated an 18.8% decrease in the total number of cases and a 37.0% decrease in age-standardized incidence. Mortality rates of sepsis demonstrated similar trends, with a 31.0% decrease in age-standardized global rates of sepsis between 1990 and 2017 [2]. A meta-analysis analyzing mortality rates in sepsis between the years 2009 and 2019 in North America, Europe, and Australia observed the 30-day mortality for sepsis being 24.4% and septic shock being 34.7%, with no significant trends in mortality identified in these years [5]. Although incidence and mortality rates of sepsis and septic shock have improved since 1990, recent data from the past decade (2009–2019) demonstrate no continuous reduction in these rates. This indicates a possible plateau in the currently available prevention and treatment options. With sepsis and septic shock mortality rates remaining a major cause of global death, further research and advancements in sepsis prevention and therapy are necessary to mitigate this global health concern. A key concern for patients with sepsis is the system-wide organ dysfunction caused by profound hypotension, notably the kidneys, which are among the first affected in a phenomenon referred to as sepsis-induced acute kidney injury (S-AKI).

Acute kidney injury is a common complication in septic patients, defined as a sudden deterioration in kidney function that leads to the buildup of waste products in the blood and disruption of fluid and electrolyte balance. Due to the fatal ramifications of this condition, the majority of patients are admitted to the hospital for close treatment and observation. Sepsis is a major cause of acute kidney injury, with 57.7% of septic patients developing S-AKI [6] and sepsis being responsible for up to 47.5% of intensive care unit (ICU) admissions for AKI [7]. Risk factors for developing S-AKI include older age and pre-existing renal disease [7], underlying liver disease, and diabetes mellitus [8]. S-AKI is strongly associated with poor outcomes. The development of S-AKI increases in-hospital mortality rates by more than threefold [8], with some studies reporting an increase in mortality up to eightfold [9]. S-AKI also significantly increases the duration of hospital stay and in-hospital mortality rate compared to non-septic AKI [10]. Table 1 summarizes the studies analyzing the rates of sepsis cases leading to S-AKI and mortality rates.

Since a major concern in sepsis mortality is S-AKI, it is important to examine the pathophysiology involved, notably the interplay of endothelial cell damage, inflammatory and oxidative stress, and coagulative dysfunction. As a result of its pathogenesis not being completely understood, treatment of S-AKI remains reactive and nonspecific. Therefore, it is essential to investigate potential therapeutic targets for this fatal condition. The renin angiotensin aldosterone system (RAAS) is an integral process in modulating responses in situations of stress, including sepsis, and promotes the pro-inflammatory and coagulative mechanisms observed in S-AKI. In this review, we will analyze the role the RAAS plays in modulating inflammatory responses and contributing to the development of S-AKI. The emerging use of RAAS inhibitors as therapeutics to minimize S-AKI will also be discussed, aiming to shed light on how the RAAS regulates the susceptibility and treatment of sepsis.

## 2. Materials and Methods

### 2.1. Protocol Design and Registration

The protocol for this study was performed based on the guidelines of the Preferred Reporting Items for Systematic Reviews and Meta-Analyses (PRISMA) [15]. The protocol for this systematic review was registered on INPLASY (Unique ID 202360098) and is available in full on the International Platform of Registered Systematic Review and Meta-analysis Protocols (INPLASY) (https://inplasy.com/inplasy-2023-6-0098/, accessed on 4 July 2023).

### 2.2. Eligibility Criteria

Inclusion criteria included randomized control trials (RCTs), cohort studies, and systematic reviews that are full-text articles in the English language (or translated into the English language) and have relevance to the previously stated relevant keywords. Human studies involved participants aged 18 years or older, from both genders, and from five continents. Studies addressing the use of RAAS inhibitors for both premorbid and therapeutic purposes in septic patients were included. Additionally, papers with a focus on sepsis-induced acute kidney injury were included.

Studies conducted on both human and animal subjects were included, since each offers an essential component to our scientific understanding. Studies utilizing animal models impose specific clinical events and examine laboratory, histological, and gross specimens. Since the use of RAAS inhibitors as a treatment for sepsis is an emerging consideration, most studies involve animal subjects to provide a greater understanding of the effectiveness and safety of this potential treatment. Human studies demonstrate trends in rates of disease development, outcomes, and medication use, and these studies were used to investigate the risk of developing S-AKI with the established use of RAAS inhibitors.

Exclusion criteria included studies that were not relevant to the research question, those that did not meet the inclusion criteria, and those with inadequate or incomplete data. Studies that were not full-text or were in a language other than English were not included. Additionally, studies that were not focused on the RAAS or the use of RAAS inhibitors were excluded to promote the assessment of direct associations.

### 2.3. Study Selection

Studies were searched for using electronic databases (Medline via PubMed, Google Scholar) from inception to May 2023. This systematic review article of current and international literature was conducted using the major online database, PubMed. Studies published in the last ten years were included, comprising those published from November 2014 to May 2023. Studies and review articles were selected using the relevant keywords “sepsis”, “sepsis-induced acute kidney injury”, “renin angiotensin aldosterone system”, “angiotensin II”, “endothelial cell damage”, “oxidative stress”, and “microthrombi formation”. Additionally, reference lists from articles were used to further search for related references.

### 2.4. Quality Assessment

A search and selection strategy was performed by two independent authors, S.T. and G.Z., and a third reviewer, H.M., evaluated the studies in cases of disagreement. The quality assessment of the cohort studies for human populations used in this systematic review was conducted using the Joanna Briggs Institute (JBI) tool. This critical appraisal checklist considers study design and protocol, internal validity and risk of bias, and data interpretation and reporting to form a score out of 11 quality criteria [16]. Quality assessment data individually analyzed by each of the authors, S.T. and G.Z., were compared and are summarized in Supplementary Table S1. SYRCLE’s Risk of Bias (RoB) tool for animal intervention studies consists of ten characteristics to assess for risk of bias, including selection bias, performance bias, and detection bias. SYRCLE’s RoB tool was used to assess the methodological quality of animal studies in this study, and two authors (S.T. and G.Z.) independently appraised these studies [17].

## 3. Results

### 3.1. Study Characteristics

**Figure 1:**
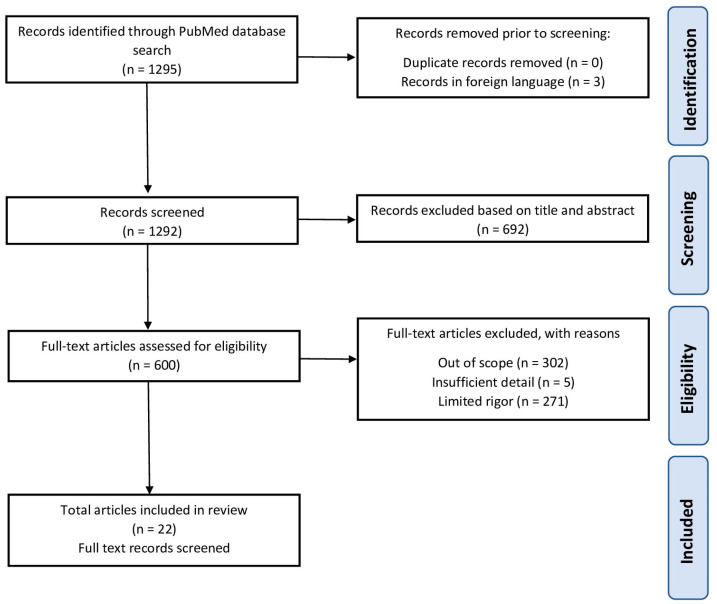
PRISMA 2020 Flow Diagram for the search and selection of studies.

A total of 1295 papers were found after searching the relevant keywords. Of these, three papers were excluded because they were not in English, and 692 articles were excluded based on title and abstract. Of the remaining articles, 302 were excluded for being out of scope, five were excluded for insufficient detail, and 271 were excluded after full-text screening. Finally, the selected 22 full-text articles fulfilled the inclusion and exclusion criteria and were included in this systematic review, in which the relevant literature was narrated in a concise account, key findings were summarized, and discrepancies and gaps were addressed. Figure 1 depicts the screening process for the inclusion of articles in this systematic review.

Using the JBI tool, the quality of the human cohort studies (n = 10) and meta-analysis reviews (n = 2) used in this review have an average score of 7.6 out of 11 quality criteria, with studies scoring 6 out of 11 quality criteria or higher. According to the JBI critical appraisal checklist for cohort studies, the quality of the included studies ranges from moderate to good. Based on SYRCLE’s RoB tool for animal studies, the animal studies used in this review (n = 10) have an average score of 7.8 out of 10 quality points.

The 22 studies included in this systematic review were published between the years 2014 and 2023, with the majority being developed in Europe (50.0%), followed by Asia (25.0%), a combination of multiple continents (16.7%), and North America (8.3%). Countries included in the studies comprise France, Greece, Taiwan, South Korea, Spain, the United Kingdom, and the United States. In most studies, participants were collected in a population-based design, and a minority focused on single-center locations in hospital centers. The number of participants in each study ranged from 297 to 549,851. Table S1 compiles participant and study characteristics.

### 3.2. Sepsis-Induced Acute Kidney Injury

S-AKI is characterized by an acute decline in kidney function in the setting of sepsis, recognized by widely available assessment tools including elevated serum creatinine and blood urea nitrogen (BUN) and decreased urine output. Hypoperfusion of the kidney is the prevailing attributable cause of S-AKI since causes of non-septic AKI (heart failure, major surgery, hypovolemia) are associated with hypoperfusion and ischemic injury known to cause acute tubular necrosis. Studies have observed decreased renal blood flow (RBF), lower renal oxygen delivery, and increased tubular injury in patients with sepsis, which supports ischemia as a leading mechanism of S-AKI [18,19]. Despite these findings, studies have also observed S-AKI in septic animals and septic humans with normal RBF [20,21,22] and in patients without a history of hypotension [12]. In light of these observations, the involvement of other mechanisms in the pathogenesis of sepsis is also at play. In addition to the classic model of hypoperfusion as a cause of S-AKI, a “unified theory” proposes an interplay of endothelial cell damage, inflammatory and oxidative stress, and coagulative dysfunction [23].

#### 3.2.1. Endothelial Cell Damage

Endothelial cells (ECs) are vascular cells that form the lining of all circulatory vessels to maintain the structural integrity of the microvasculature, regulate blood flow at a microvascular level, maintain a permeable layer between the intravascular lumen and extracellular space, and secrete signals to communicate with adjacent connective tissue [24]. The activation of ECs is believed to contribute to the pathogenesis of sepsis-induced acute lung injury due to profound pulmonary vascular leakage and interstitial edema [25]. A similar mechanism is likely to play a role in S-AKI. In the kidney, ECs line arterioles, post-capillary venules, glomerulus, and peritubular capillaries and work alongside podocytes and mesangial cells to support filtration functions and regulate blood pressure in the kidney’s microvasculature [24,26].

The glycocalyx layer, a gel-like surface consisting of glycosaminoglycans and proteoglycans that coats healthy endothelium, maintains vascular integrity and leukocyte trafficking [24]. In a septic state, this layer is observed to have reduced thickness and evidence of destruction, likely as a result of pro-inflammatory cytokines and chemokines [27,28]. In addition to the breakdown of the glycocalyx layer, an inflammatory state also interferes with cadherin proteins, key components of adherens junctions, disrupting endothelial intercellular permeability. The resulting vascular leakage promotes edema and extravasation of leukocytes into extravascular compartments, interfering with normal cell function in several organs. In the kidneys, a study found that elevated levels of glycocalyx component proteins, such as vascular endothelial cadherin (VE-cadherin), and endothelial junction components in the circulation likely indicated a breakdown of these structures and were associated with more severe cases of S-AKI [29].

ECs readily react to environmental changes due to their containing Weibel-Palade bodies and storage granules for proteins such as von Willebrand Factor (vWF) and Angiopoietin 2 (Angpt2) [26]. The interaction between platelets and vWF on endothelial surfaces activates the Tie2 tyrosine-kinase receptor on endothelial cells with the downstream secretion of Angpt2. The induction of the Tie2-Angpt2 system by vWF was observed to have a role in reducing diapedesis-associated microvascular leakage [30]. This anti-inflammatory function is crucial in mitigating tissue damage in the setting of infection or tissue repair. ECs also express inflammatory adhesion molecules, including E-selectin, vascular cell adhesion protein (VCAM1), and intercellular adhesion molecule 1 (ICAM1), in response to increased levels of the inflammatory cytokines IL-6 and IL-8 [3]. The upregulation of VCAM1 and ICAM1 promotes leukocyte recruitment to the endothelial surface and extravasation into the extravascular spaces. In the pathogenesis of sepsis, the anti-inflammatory efforts exerted by the Tie2-Angpt2 system are likely overwhelmed by inflammatory cytokines, resulting in leukocyte leakage and edema.

#### 3.2.2. Inflammatory and Oxidative Stress

During sepsis, the abundant presence of PRRs in the circulation activates PAMPs, which initiate a downstream cascade resulting in the release of pro-inflammatory mediators, including IL-1, IL-6, and TNF-α. Toll-like receptors (TLR), a type of PAMP, are present in many organs, including renal tubular epithelial cells (TECs). When activated during sepsis, renal TECs exhibit increased production of pro-inflammatory cytokines and reactive oxygen species (ROS), both of which directly damage renal tubular cells and activate the pro-inflammatory macrophage phenotype (M1) [31]. The overabundance of circulating cytokines causes direct tubular injury and death, interferes with the function of normal structural proteins, and leads to the paracrine release of maladaptive repair factors from TECs [32]. The pathology of kidney biopsies in patients who had succumbed to septic shock demonstrated tubular cell apoptosis and renal tubular casts [33], which obstruct renal tubules and prevent appropriate filtration functions. On the other hand, autophagy of renal TECs may also be considered a protective effort to remove damaged cellular components and maintain homeostasis, as it has been observed to alleviate inflammation and renal dysfunction in animal models of S-AKI [34,35]. A recent study demonstrated that inducing autophagy of renal endothelial cells in septic mice alleviated glomerular injury but had no significant effect on renal function [36]. These findings may indicate a role in renal endothelial stability in early S-AKI, and excess autophagy response in later stages of sepsis leads to deterioration in renal function.

The abundance of ROS during sepsis risks damaging cellular DNA, proteins, and lipid structures. Although the exact mechanisms for mitochondrial dysfunction during sepsis are incompletely understood, damage by ROS is likely a major cause due to the high susceptibility of mitochondrial DNA and proteins to oxidative stress. A study found patients with S-AKI had excess mitochondrial DNA damage, lower mitochondrial mass, and upregulated mRNA expression of oxidative damage markers. Impairments of mitochondrial function led to ATP depletion due to cytochrome c damage and reduced antioxidant defenses, forming a vicious cycle that leaves the cell vulnerable to more damage by ROS [37]. The consequential reduction in antioxidants such as glutathione, ATP availability, and the cell’s reduced ability to utilize oxygen leads to damage and dysfunction at the cellular and organ levels [38]. Changes in mitochondrial permeability and the release of cytochrome c into the cell trigger the caspase cascade and apoptosis, further contributing to cellular death [39].

Nitric oxide produced by the endothelium (eNO) is a basal regulatory vasodilator in renal blood flow hemodynamics. The inducible form of nitric oxide (iNO) is dramatically produced by the endothelium in situations of stress to maintain blood flow and counteract vasoconstriction by endothelin, norepinephrine, and angiotensin II. The actions of iNO are abnormally distributed, with a greater vasodilatory effect in the renal cortex, shunting blood away from the medullary and peritubular regions of the nephron [40]. Inequality in renal microcirculation leaves the aforementioned areas susceptible to ischemia. Additionally, nitric oxide itself has an unpaired electron and is a free radical, with byproducts such as peroxynitrite and other ROS contributing to oxidative stress in the mitochondria. These byproducts use and deplete antioxidants in the mitochondria, further contributing to the previously mentioned cycle of ROS-induced ROS damage [39]. In a study on septic rats, inhibiting iNOS led to a reduction in oxidative stress and decreased markers of organ damage [41].

#### 3.2.3. Coagulative Dysfunction

The balance of anticoagulant and procoagulant factors is crucial in preventing microthrombi formation and ensuring appropriate blood flow, and there are many modulators involved in maintaining this circulatory homeostasis. Endothelial thrombomodulin (TM) is a prominent anticoagulant, binding to thrombin and preventing its interaction with platelets and coagulation factors. TM also binds and activates protein C, which degrades coagulation factors. During sepsis, the vascular endothelium is damaged by pro-inflammatory and oxidative factors. As previously mentioned, the glycocalyx layer of the endothelium is crucial in maintaining a permeability barrier and preventing excess leakage into extravascular spaces. The glycocalyx layer also plays a role in maintaining coagulative homeostasis by anchoring TM and antithrombin to the endothelial surface via glycosylamines [42]. Breakdown of the glycocalyx layer due to inflammation and oxidative stress disrupts the functions of these major anticoagulant modulators. Additionally, endothelial cell damage leads to the release of vWF, which binds to and initiates platelet aggregation, leading to the activation of the coagulation cascade.

The overwhelming amount of inflammatory mediators such as neutrophils, monocytes, and platelets are believed to contribute to the development of thrombus, a phenomenon referred to as “immunothrombus”. When encountering a pathogen, neutrophils release extracellular traps (NETs) containing DNA, histones, granule proteins, and other prothrombotic mediators. The interaction between NETs, platelets, and endothelial cells contributes to sepsis-induced coagulopathy [43].

In septic rats, renal levels of procoagulant factors such as thrombin, fibrin, and tissue factor were elevated compared to non-septic cases. Subsequent fibrin deposition in glomeruli and renal arterioles increased creatinine and BUN [44]. Emerging studies have observed that coagulative disorders, including thrombocytopenia, elevated international standardized ratio (INR), prolonged partial thrombin time (PTT), and prolonged prothrombin time (PT), were correlated with worse outcomes in patients with S-AKI [45,46].

Disruption of the balance of coagulative processes incites a major phenomenon seen in sepsis: disseminated intravascular coagulation (DIC). DIC is characterized by systemic microthrombi formation, leading to vessel blockage and organ ischemia, and exhaustion of procoagulant factors, resulting in internal bleeding. Recombinant TM treatment in septic patients with severe coagulopathy demonstrated improvements in 28-day and in-hospital mortality [47], although similar studies failed to show beneficiary findings [48]. In septic mice, levels of TM and endothelial protein C receptor (EPCR) decreased by 50% within 12 h of the initiation of sepsis [49]. Inducing the expression of TM and EPCR in the kidneys of septic rats exerted anticoagulant effects and attenuated kidney injury [50].

**Figure 2:**
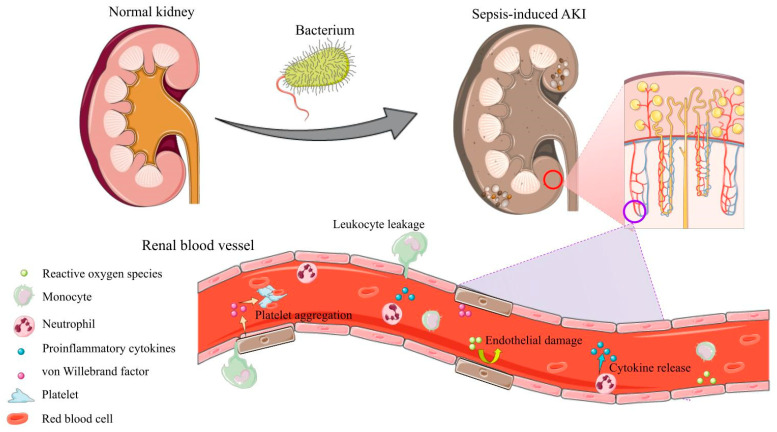
Interplay of Mechanisms in the Development of S-AKI. This figure displays the abundance of pro-inflammatory and coagulative factors present in the renal microvasculature during sepsis. These elements migrate from both systemic and local sources. Endothelial damage by ROS* and cytokines exposes vWF*, which activates platelet aggregation and the coagulation cascade. Having also migrated to the renal circulation, leukocytes, including monocytes and neutrophils, release pro-inflammatory cytokines and leak into extravascular spaces, causing edema. The interplay of these processes contributes to the pathogenesis of S-AKI. *ROS: reactive oxygen species; vWF: von Willebrand Factor. This figure was partly generated using Servier Medical Art, provided by Servier and licensed under a Creative Commons Attribution 3.0 unported license.

The exact mechanism by which sepsis causes AKI is not completely understood but likely includes an interplay of several processes, including endothelial cell damage, inflammatory and oxidative factors, and microthrombi formation, as portrayed in Figure 2. Since the integration of several host systems is involved in the pathophysiology of sepsis, the treatment of S-AKI is nonspecific and reactive. As a fundamental system involved in the stress response, the RAAS is a key network with several interplaying factors critical to regulating blood pressure, coagulation, and oxidative stress. RAAS likely plays a critical role in the pathophysiology and host responses to sepsis and S-AKI.

### 3.3. Renin Angiotensin Aldosterone System

The RAAS is an intricate hormonal system that functions to regulate blood pressure and maintain fluid balance. The RAAS is initiated when a decrease in blood volume or blood pressure is detected by baroreceptors in the afferent arteriole of the kidney or when a low sodium concentration is sensed by macula densa cells in the cortical thick ascending of the renal tubule. These homeostatic imbalances trigger the release of the enzyme renin from the nearby juxtaglomerular cells, located between the distal convoluted tubule and afferent arteriole in each nephron unit of the kidney. The RAAS is also activated through the sympathetic nervous system as a vital response to low blood pressure. In the sympathetic response, norepinephrine binds to the beta-adrenergic receptors located on juxtaglomerular cells, triggering renin release.

Angiotensinogen (AGT) is a precursor protein that is predominantly produced and secreted by hepatocytes and in minor quantities by adipose tissue, the heart, and the brain [51]. AGT is maintained in abundant concentrations in the bloodstream as a precursor protein for angiotensin I and II [52]. The cleavage of AGT by renin releases angiotensin I and acts as the rate-limiting step of the RAAS cascade. 

Angiotensin I is an inactive protein of the RAAS and is converted to the active protein angiotensin II by the Angiotensin-Converting Enzyme (ACE). ACE catalyzes the conversion of angiotensin I to angiotensin II by protein cleavage [53]. ACE is primarily found on the surface of vascular endothelial cells in the lungs, as well as in minor quantities in the kidneys and testes. The function of ACE is not limited to the RAAS, as it acts to break down bradykinin, a peptide known for its role in mediating pain and inflammation. ACE produces the key protein of the RAAS, angiotensin II, which leads to multiple downstream effects that ultimately result in increased blood pressure and blood volume while restoring electrolyte imbalance through sodium and water reabsorption.

Angiotensin II is regarded as a potent vasoconstrictor of vascular smooth muscles in kidney arterioles and directly increases sodium absorption in the proximal convoluted tubule by binding to the Angiotensin II Type 1 Receptor (AT1-R) and increasing the activity of the Na+/H+ antiporter. Angiotensin II also stimulates the release of the mineralocorticoid Aldosterone from the zona glomerulosa of the adrenal cortex, mediated through upregulation of Aldosterone Synthase. Sodium is absorbed from the renal collecting duct through the epithelial sodium channels (ENac) located on the apical surface of the principal cells. Aldosterone binds to the mineralocorticoid receptor (MR) on the principal cells and increases the activation of these ENac channels and sodium absorption. Aldosterone acts to increase the activity of the Na+/K+ ATPase on the basolateral membrane, resulting in a net increase in sodium reabsorption and potassium secretion. Aldosterone regulates salt and water homeostasis through neural effects as well, by binding to the MRs located in the brain and increasing thirst and salt appetite [54].

Apart from its effects on the renal system, angiotensin II has a significant role in increasing blood volume and pressure indirectly through its neural effects. Angiotensin II acts on the hypothalamus to induce a sensation of thirst, leading to increased fluid consumption, resulting in blood volume expansion and consequently the regulation of blood pressure. Angiotensin II also increases the secretion of Antidiuretic hormone (ADH) from the posterior pituitary. ADH acts on vasopressin receptors to increase circulating fluid volumes by increasing water reabsorption through the transcription and insertion of Aquaporin 2 channels in the apical surface of the distal convoluted tubule and collecting duct of the kidney.

To prevent excess activation of the RAAS, a negative feedback loop is initiated once blood volume, blood pressure, and electrolyte balance have been restored. This negative feedback loop revolves around suppressing renin release through multiple mechanisms. One primary mechanism is the suppression of renin release from juxtaglomerular cells in response to elevated concentrations of angiotensin II. Similarly, increased concentrations of aldosterone result in the suppression of renin in the adrenal glands. Negative feedback is also implemented once the renal vasculature senses elevated blood pressure, resulting in decreased renin secretion from the juxtaglomerular cells. Factors disrupting the RAAS negative feedback loop, such as hypertension (HTN), heart failure, diabetes mellitus, and CKD, can lead to the worsening of health conditions and further disease progression.

### 3.4. Role of the RAAS in S-AKI

#### 3.4.1. Involvement of Angiotensin II

Sepsis-induced vasodilation leads to systemic hypotension and decreased kidney perfusion, activating the RAAS. As mentioned earlier in this paper, angiotensin II is a crucial contributor and modulator of restoring hemodynamic and electrolyte balance through vasoconstriction and its downstream effects through the RAAS. While critical in maintaining blood pressure, in cases of RAAS overactivation, such as in S-AKI, angiotensin II has detrimental effects on kidney function. Overactivation of angiotensin II induces a pro-inflammatory and prothrombotic environment further exacerbating damage to renal tissue, tubules, and vasculature in cases of disease such as sepsis.

Angiotensin II upregulates the expression of known pro-inflammatory mediators such as TNF-α and IL-6, which recruit immune cells to the glomerulus and interstitium, leading to inflammatory damage in the kidney [55]. The expression and secretion of ICAM have also been shown to be upregulated by angiotensin II via AT1-R [56], allowing for increased migration of immune cells and further tissue damage. Apart from its direct recruitment of inflammatory cells, angiotensin II activates the transcription factor Nuclear Factor-kappa β (NF-kβ) through the Angiotensin II Type 2 Receptor (AT2-R) [57]. The activation of NF-kβ promotes the expression of pro-inflammatory genes and further exacerbates renal inflammation. A study on septic mice observed that the administration of angiotensin (1-7) [Ang-(1-7)] suppressed the activation of NF-kβ, reduced levels of pro-inflammatory cytokines and oxidative factors, and inhibited the downstream activities of angiotensin II, and these findings were correlated with alleviation of renal injury [58]. In addition to activating NF-kβ, studies have shown that angiotensin II also plays a more direct role in the oxidative damage of renal cellular components through the intracellular formation of ROS such as hydrogen peroxide and superoxide anion. These ROS are transducers of cell growth, apoptosis, and cell migration and affect the expression of inflammatory and extracellular matrix genes [59].

Another critical mechanism in the pathogenesis of S-AKI is coagulative imbalance. Angiotensin II has prothrombotic effects on renal circulation that may contribute to the formation of microthrombi and overactivation of the coagulative cascade. Angiotensin II’s prothrombotic effects on large vessels are mediated through the activation of AT1-R and upregulation of aldosterone. The inhibition of ATR1 and the administration of Valsartan, an ARB, were observed to reduce stasis-induced venous thrombotic changes by improving bleeding time, platelet adhesion, and levels of nitric oxide [60]. The prothrombotic environment induced by angiotensin II also impacts arteriole microcirculation by activating AT2-R involved in thrombus initiation and the Angiotensin II Type 4 Receptor (AT4-R) involved in thrombus stabilization. Both AT2-R and AT4-R appear to interact with bradykinin-1 and endothelin-1A receptors to mediate microvascular thrombus formation [61]. ACE, the enzyme catalyzing the production of angiotensin I to angiotensin II, also plays a role in creating a prothrombotic state by breaking down bradykinin, which acts as a stimulant to tissue plasminogen activator. This prothrombotic state induced by angiotensin II contributes to renal damage through vascular occlusion and ischemic damage of the renal microvasculature, a key feature observed in S-AKI.

#### 3.4.2. Use of RAAS Inhibitors

**Figure 3:**
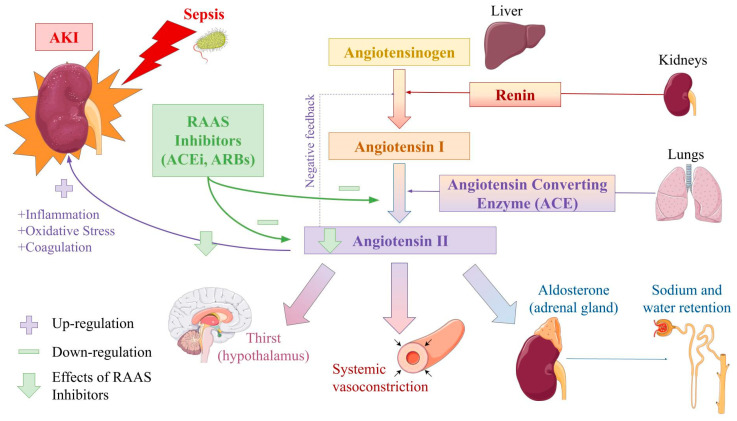
Role of the RAAS* and RAAS Inhibitors in the Pathophysiology of S-AKI. This schematic representation of the renin angiotensin aldosterone system conveys its key components and main effects on the human body. The role of angiotensin II in inflammation, oxidative stress, and coagulation, which contribute to the development of S-AKI, is also indicated. RAAS inhibitors minimize these processes by modulating ACE* activity and blocking angiotensin II receptors. *RAAS: renin angiotensin aldosterone system; ACE: angiotensin converting enzyme. This figure was partly generated using Servier Medical Art, provided by Servier and licensed under a Creative Commons Attribution 3.0 unported license.

The fundamental involvement of the RAAS in blood pressure control, remodeling of cardiac and kidney tissues, and inflammation makes it a key target for treatments of various pathologic processes, including systemic HTN, congestive heart failure (CHF), and CKD. RAAS antagonists, notably Angiotensin Converting Enzyme Inhibitors (ACEi) and Angiotensin Receptor Blockers (ARBs) are extremely beneficial in the control of cardiac and renal pathologies and are considered first-line treatment for uncomplicated HTN, HTN with CHF, previous myocardial infarction (MI), and CKD [62]. In the kidney, RAAS inhibitors reduce glomerular pressure and proteinuria and slow the progression of kidney fibrosis. These protective efforts are mainly exerted by limiting the effects of angiotensin II on efferent arteriole vasoconstriction and reducing the production of cytokines such as transforming beta-factor (TGF-β), which contribute to glomerular fibrosis [63]. In light of the aforementioned angiotensin II-induced inflammation and coagulopathy, the RAAS is likely a significant player in the pathogenesis of sepsis and S-AKI and provides the possibility for targeted therapy. The RAAS, the effects of angiotensin II on the body and S-AKI, and the role of RAAS inhibitors in the pathogenesis of S-AKI are conveyed in Figure 3.

On the other hand, RAAS inhibitors are used to treat HTN, lowering systemic blood pressure by mitigating the effects of angiotensin II on vascular tone and aldosterone. This feature may exasperate a major hallmark of sepsis: profound hypotension. Therefore, it is important to differentiate between the chronic use of ACEi/ARBs and the use of RAAS antagonists as therapy during sepsis. Since ACEi/ARBs lower blood pressure, the onset of sepsis after their chronic use may put these patients at risk for worse outcomes due to the inhibition of the RAAS from responding to hypotension. Alternatively, the acute use of RAAS inhibitors in the setting of sepsis may not immediately affect blood pressure, as the downstream effects of ACEi/ARBs require several hours and instead provide remediation for the oxidative, inflammatory, and coagulopathic effects of the RAAS, leaving time for interventions addressing blood pressure [64]. Essentially, the use of ACEi/ARBs as risk factors or as a treatment for sepsis should be assessed independently of one another. Studies analyzing the consequences of both established and hospital-only (or postadmission) use of RAAS antagonists are crucial in understanding the potential therapy these medications provide for sepsis and S-AKI.

#### 3.4.3. Impact of RAAS Inhibitors on Risk for S-AKI

The effect of preadmission and chronic use of ACEi/ARBs on the rate of sepsis mortality has resulted in conflicting findings in several studies. Many studies have observed decreased short-term mortality risk after sepsis with the use of established RAAS inhibitor treatment for both ACEi and ARBs [65,66,67]. Other studies found a very limited or no association between prior use of ACEi/ARBs and mortality after sepsis [68]. Finally, some studies found that the preadmission use of ACEi/ARBs was correlated with an increased risk of developing sepsis [69]. Whether the established use of RAAS antagonists is protective or harmful for the development and outcomes of sepsis, further research is needed in light of inconsistent and contradictory findings.

Current analysis examining RAAS inhibitor use as a risk factor with regard to S-AKI similarly provides mixed conclusions. One study observed a higher risk of AKI for emergency medical admissions in patients using ACEi/ARBs compared to non-AKI admissions [70]. A meta-analysis assessing a large cohort of septic patients found several characteristics significantly associated with increased risk of S-AKI; however, these risk factors vary greatly, including underlying comorbidities such as diabetes mellitus and HTN, use of ACEi/ARBs and diuretics, infection source, and microbial type. Due to the high probability of confounding variables and stronger associations with certain variables, further research is required before forming a direct correlation between using RAAS inhibitors and the risk of S-AKI [71]. A similar retrospective study with a large study size identified consistent trends; with the use of ACEi/ARBs being associated with a slightly increased risk of S-AKI, but individual patient characteristics and cases playing a larger role [72]. Further studies have observed corresponding findings [73,74,75]. As previously mentioned in this paper, these findings suggest the established use of ACEi/ARBs prior to the onset of sepsis exacerbates hypotension and promotes AKI in the setting of sepsis.

Given that the RAAS is predominantly responsible for responding to cardiovascular changes to maintain appropriate blood pressure, inhibiting this system may give rise to exasperated hypotension during sepsis. For this reason, it is suitable to compare differences in blood pressure during sepsis with regard to those with and without prior use of ACEi/ARBs. A study assessing the risk for AKI in emergency medical admissions observed that of all patients with prior ACEi/ARBs use, patients with significantly lower systolic blood pressure were more at risk of developing AKI [70]. Another study found that mean arterial pressure (MAP) was significantly lower in septic patients who developed AKI compared to those who did not. When comparing patients with and without ACEi/ARB use, MAP was lower in the group with prior ACEi/ARB use, although not significantly [73]. Patients with established use of ARBs in the higher MAP group had significantly less severe S-AKI compared to patients with established use of ARBs in the lower MAP group [76].

The RAAS is critical for regulating blood pressure and blood osmolality. Angiotensin II and aldosterone mediate these effects via vessel vasoconstriction and sodium retention. Activation of the RAAS is triggered by the autonomic nervous system in periods of stress, low blood pressure, or low blood osmolality. During sepsis, the prior use of RAAS inhibitors presents a concern due to possible exacerbations of hypotension and interference with the normal physiologic mechanism to modulate blood pressure. Although studies agree on the correlation between the established use of RAAS inhibitors and an increased risk of S-AKI, their findings vary in strength. Due to heterogeneity in the strength of associations, individual patient characteristics are likely more predominant in determining the risk of developing S-AKI than the independent use of RAAS antagonists. In light of the study findings in this paper, the prior use of RAAS inhibitors does not independently increase or decrease the risk of developing AKI in the setting of sepsis; rather, the individual characteristics of the patient are more likely to play a role. The presence of underlying comorbidities in the patient may hinder proper blood pressure management, and ACEi/ARB therapy intensifies this phenomenon. In several studies [70,71,72,73,74,75], patients with underlying comorbidities on RAAS inhibitor therapy had an increased risk for S-AKI compared to those without underlying comorbidities on RAAS inhibitor therapy. This suggests that RAAS inhibitors have a role in aggravating existing conditions and advancing hypotension. To minimize the hypotensive ramifications of RAAS inhibitor therapy, recommendations to physicians include adjusting medication frequency and dose according to estimated Glomerular Filtration Rate (eGFR) and withholding these medications during times of acute illness [70].

On the other hand, key components in the RAAS, especially angiotensin II, contribute to the pro-inflammatory damage and coagulopathy observed in the pathogenesis of S-AKI. Therefore, a balance between hypotensive, anti-inflammatory, and anticoagulative effects in the context of sepsis and S-AKI allows RAAS inhibitor therapy to be a possible therapeutic target in treating these conditions.

#### 3.4.4. Impact of RAAS Inhibitors on Outcomes of S-AKI

RAAS inhibitors are an emerging therapy for sepsis due to their ability to decrease inflammatory and oxidative stress, endothelial damage, and microthrombi formation. As initially proposed in 2010, using RAAS inhibitors as a treatment for sepsis may help attenuate these processes and limit organ failure and mortality [77]. The essential point of consideration for using RAAS antagonists is minimizing hypotensive effects and optimizing anti-inflammatory and anticoagulative results.

Several studies have focused on analyzing the impact of RAAS antagonist therapy on sepsis-induced cardiac damage. Removing the AGT gene from the liver of mice reduced levels of angiotensin II and alleviated sepsis-induced myocardial dysfunction. A therapy targeting AGT or its derivatives, angiotensin I and II, would have a similar effect on the Ang-II-dependent pathway described in the study [78]. Given that levels of angiotensin II and Ang-(1-7) were elevated in patients with sepsis-induced cardiomyopathy (SIC) compared to non-SIC septic patients, a study examined the rates of SIC in septic mice treated with Losartan, an ACEi [79,80]. Septic mice treated with losartan had decreased areas of myocardial tissue damage, myofibril disorder, and interstitial inflammation in comparison to septic mice with no treatment. Labs of losartan-treated mice demonstrated decreased brain natriuretic peptide (BNP), pro-inflammatory cytokines IL-1, IL-6, TNF-α, and Ang II levels. Interestingly, the cardiac histology of losartan-treated mice showed reduced levels of ROS and increased levels of ATP, and they had a longer survival period, suggesting a protective role against oxidative stress and mitochondrial damage. Similar improvements were also observed in the spleen of these mice, supporting losartan as a therapy with systemic organ protection [79]. The use of a renin inhibitor, aliskiren, as an adjunct therapy in septic mice, increased levels of glutathione and decreased levels of ROS and pro-inflammatory cytokines, including IL-6 and TNF-α. The lung tissues of these mice demonstrated protection from oxidative stress and inflammatory modulators associated with higher doses of aliskiren [81]. A similar study using a dual inhibitor of neprilysin and ACE on septic mice found consistent results, including decreased levels of pro-inflammatory cytokines and evidence of lung protection on histology [82]. Similar findings were seen using Sacubitril/valsartan, a dual inhibitor of neprilysin, and an ARB [83]. The inhibition of the RAAS, whether by gene knockout or the use of an antagonist, demonstrated improvements in several organs, including the heart, spleen, and lungs, and improved overall survival.

Since the kidneys are significantly affected by sepsis and its hallmarks of inflammatory and oxidative damage, RAAS antagonists may be applied to S-AKI in correspondence with the previous principles. Septic rats treated with Ramipril, an ARB, or losartan had improved creatinine and reduced amounts of histopathological renal damage, including the maintained architecture of renal cortices, Bowman’s space, tubular dilation, and interstitial edema in comparison to septic rats without treatment. The use of RAAS inhibitors ramipril or losartan was also observed to reduce levels of angiotensin II, IL-6, TNF-α, and several ROS and overall improve survival in treated rats [84]. A similar study showed consistent findings with septic mice treated with Captopril, an ACEi, having decreased pro-inflammatory cytokines, creatinine, and ROS, in addition to improved renal function and attenuated oxidative damage in renal tissue [85]. Treatment of septic mice with Perindopril, an ACEi, demonstrated decreased loss of brush border structures in the proximal and distal tubular epithelium, lower inflammatory markers in kidney tissue, and modulated rates of apoptosis in renal cells in comparison to untreated septic mice [86].

The use of RAAS antagonists as a therapeutic option in sepsis has recently been considered, with emerging studies that help form a greater understanding of the role of RAAS inhibitors in sepsis. Several studies observed decreased rates of S-AKI, renal tissue damage, and levels of pro-inflammatory cytokines and oxidative stress with the use of RAAS antagonists as treatment during sepsis [84,85,86], as well as evidence of their playing a protective role in other organs, including the lung [81,82,83], heart [78,79,80], and spleen [79]. RAAS inhibitors modulate the activity of angiotensin II and minimize its downstream effects on inflammation and coagulation. The suppression of these processes prevents direct damage to renal TECs, ischemia secondary to microthrombi formation, and edema due to leukocyte extravasation. Although one study found that using angiotensin II as a treatment for septic mice had beneficial results on kidney function and that administration of losartan reversed this protection, the limited size of the study sample should be considered [87]. These observations were likely due to the role of angiotensin II in modulating blood pressure and ameliorating the profound hypotension that occurs during sepsis. An important consideration of using RAAS antagonists as treatment is minimizing the hypotensive ramifications while attaining the anti-inflammatory and anti-thrombotic benefits of these medications. Further studies may shed light on how to achieve this critical balance and explore RAAS inhibitor use as a treatment for sepsis in humans.

## 4. Discussion

Sepsis is a global health concern with a high incidence and mortality rate. This life-threatening condition poses a danger to every organ in the body, most notably the kidney. The pathogenesis of S-AKI is likely an interplay of several mechanisms, including ischemia due to hypotension, endothelial damage, inflammatory and oxidative stress, and coagulative imbalance. The RAAS plays an active role in regulating blood pressure and responding to situations of stress, such as sepsis. A key component of the RAAS, angiotensin II, is pro-inflammatory and procoagulant, therefore, inhibition may be beneficial in septic patients. In this review, we discussed the pathophysiology of RAAS and the development of S-AKI. The use of RAAS antagonists to minimize sepsis-induced organ damage and AKI may provide an emerging therapeutic option for this deadly condition.

The evidence found in determining if the established use of RAAS inhibitors influences the risk of developing acute kidney injury during sepsis. Findings in this study indicate that RAAS inhibitors do not independently place these patients at higher risk for developing S-AKI. Rather, these medications likely aggravate the hypotensive response in patients with preexisting cardiovascular comorbidities, such as HTN, CKD, or diabetes mellitus. Alternatively, RAAS inhibitors are used to treat cardiovascular conditions. Their use indicates patients using RAAS inhibitor medications, most notably ACEi and ARBs, have underlying conditions that are known independent risk factors for sepsis and S-AKI [4]. Adjusting for these confounding variables proves difficult, as the majority, if not all, of patients using RAAS inhibitors have these conditions; therefore, an optimal control cohort focused on RAAS inhibitors is challenging to obtain. A study analyzing chronic RAAS inhibitor users without the existence or history of underlying comorbidities acting as the control group may shed more light on this topic.

The existing treatment for S-AKI is reactive and nonspecific, warranting research into the mechanisms of pathogenesis and therapeutic considerations for this fatal condition. Studies on the use of RAAS inhibitors are an emerging topic of attention with promising findings. RAAS inhibitors are observed to have a protective effect on sepsis-induced injury of the lungs, heart, and spleen. Studies on the kidneys show the use of RAAS inhibitors decreases the risk of developing S-AKI, reduces levels of inflammatory and oxidative factors in the kidney, and decreases damage to renal tissue. An area of concern for their use as a treatment for sepsis is their hypotensive consequences. To maximize anti-inflammatory and anticoagulant effects and minimize hypotensive ramifications, large doses of RAAS inhibitor medications should be avoided. Instead, small and slow-releasing administrations of RAAS inhibitors may offer an encouraging balance between the beneficial and detrimental effects of these medications. The research focused on exploring varying pharmacokinetics may offer a greater understanding of the potential therapeutic role of RAAS inhibitors.

It Is essential to note the heterogeneity in the strength of outcomes of studies assessing the established use of RAAS inhibitors in sepsis and S-AKI. Although studies were consistent in reporting a direct correlation between the risk of developing S-AKI and the use of RAAS inhibitors, there was variability in the strength of the correlation [70,71,72,73,74,75]. Considering the large sample sizes in these studies and varying correlation strengths, it seems reasonable to inspect differences in setting characteristics, such as the country of study impacting patient group characteristics, expected severity of sepsis, and resource availability in treating infections and/or sepsis. Additionally, despite the large sample size in each study, the overall number of articles on this topic is limited and warrants further investigation to apply findings to a population-size scale.

This review uses many studies with animal subjects to assess the therapeutic potential of RAAS inhibitors. Animal studies do not consider the existence of underlying comorbidities, confounding factors in humans, or differences in physiology. Therefore, this systematic review only recognizes the potential for the use of RAAS inhibitors as an emerging therapy and does not identify their use as conclusive evidence for clinical use. The direct comparison of study findings was not numerically analyzed, preventing the identification of significance from pooling numerical findings. Finally, there may be a possible bias that cannot be excluded due to overlapping cohorts between the included studies.

## Figures and Tables

**Table 1:** Summary of Sepsis Cases Leading to Acute Kidney Injury and Mortality.

Study	AKI * Status of Septic Patient	Number of Patients	Median Age (in years)	Male n (%)	Overall Rate of S-AKI * in Septic Patients	Overall Mortality Rate n (%)
Suh 2013 [7]	non-AKI	419	58.7 ± 18.3	195 (46.5)	57.70%	12 (2.9)
	AKI	573	66.8 ± 14.3	310 (54.1)		94 (16.4)
Hsu 2019 [8]	non-AKI	597	68.3 ± 15.5	366 (61.3)	14.22%	127 (21.3)
	AKI	99	68.8 ± 16.1	63 (63.6)		71 (71.7)
Hoste 2003 [11]	non-AKI	155	53	106 (68.4)	16.20%	44 (28.4)
	AKI	30	62	20 (66.6)		17 (56.7)
Murugan 2010 [12]	non-AKI	1205	65.2 ± 17.1	634 (52.6)	34.37%	16 (1.3)
	AKI	631	73.4 ± 14.5	320 (50.7)		70 (11.1)
Poukkanen 2013 [13]	non-AKI	430	63.0 ± 11.0	285 (66.3)	53.15%	18.1 (78)
	AKI	488	66.0 ± 10.0	305 (62.1)		29.3 (143)
Bu 2019 [14]	non-AKI	90	63.54 ± 17.46	49 (54.4)	59.46%	9 (10.00)
	AKI	132	65.70 ± 16.92	74 (56.1)		55 (41.67)

* AKI: acute kidney injury; S-AKI: sepsis-induced acute kidney injury.

## Data Availability

No new data were created or analyzed in this study. Data sharing is not applicable to this article.

## Supplementary Materials

The following supporting information can be downloaded at: https://www.mdpi.com/article/10.3390/jcm12144566/s1, Table S1: Descriptive table of study and participant characteristics; quality ratings.
