# Supplementary material for: Role of the Renin Angiotensin Aldosterone System in the Pathogenesis of Sepsis-Induced Acute Kidney Injury: A Systematic Review
Source: J Clin Med. 2023 Jul 8;12(14):4566. doi: 10.3390/jcm12144566 (PMC10380384; doi:10.3390/jcm12144566)
Supplement: Supplementary file 1 [file jcm-12-04566-s001.zip › jcm-2448134-supplementary.pdf]

First Author and Year	Study Design	Study Location	Cohort Evaluated *(N)*	Source of Data Collection	Quality of Study per JBI Tool
Hsieh, 2020	Population-based retrospective observational	Taiwan	N = 33,213	Retrieved from database of a sepsis cohort between 1999 and 2013 from the National Health Insurance Research Database (NHIRD) of Taiwan	8/11
Lee, 2021	Population-based retrospective observational	South Korea	N = 27,628	Retrieved from Health Insurance Review and Assessment Service (HIRA) annual claims data of the total population in South Korea from 2009 to 2013	6/11
Hsu, 2020	Population-based retrospective observational	Taiwan	N = 52,727	Retrieved from National Health Insurance Research Database of Taiwan on patients hospitalized with sepsis who were longitudinally followed from January 2001 to December 2011	9/11
de Roquetaillade, 2020	Single-center retrospective observational	France	N = 735	Data collected on adult patients (age ≥18 years old) diagnosed with septic shock within the first 48 h of ICU admission in 24-bed tertiary medical intensive care unit (ICU) between 2008 and 2016	9/11
Dial, 2014	Population-based retrospective cohort design	United Kingdom	N = 549,851	Data retrieved from the UK General Practice Research Database between 1 January 2000 and 30 June 2009	7/11
Feidakis, 2021	Single-center retrospective observational	Greece	N = 577	Retrieved from Emergency Room (ER) who were subsequently admitted to the Division of Medical Services at Attikon University Hospital in Athens, Greece, between June and July 2018	6/11
Liu, 2020	Systematic Review	Several (18 countries, 4 continents)	N = 55,911	Systematic literature search was performed in Medline, Embase, Cochrane Library, PubMed, and Web of Science, databases from inception to June 2019 with no restrictions	8/11
Mansfield, 2016	Population-based cohort	United Kingdom	N = 570,445	Data obtained from UK primary care practices contributing to the Clinical Practice Research Datalink (CPRD) eligible for linkage to hospital records data from the Hospital Episode Statistics (HES) database between April 1997 and March 2014	7/11
Suberviola, 2017	Single-center prospective observational	Spain	N = 386	Data collected from the intensive care department at Marques de Valdecilla University Hospital in Spain between September 2005 and August 2010	8/11
Flannery, 2022	Single-center retrospective observational	United States	N = 707	Retrieved from a tertiary care, academic medical center consisting of inpatient admissions from January 1, 2013 to July 31, 2020	9/11

Hasegawa, 2022	Systematic Review	Several	N = 96,159	Systematic literature search was performed on 2/12/2022 via MEDLINE, the Cochrane Central Register of Controlled Trials, and Embase	8/11
Demiselle, 2021	Population-based cohort	France	N = 297	Data included in The Sepsis and Mean Arterial Pressure (SEPSISPAM) trial. SEPSISPAM trial was a multicenter, randomized, open label, controlled trial	7/11
